# PSMs of Hypervirulent *Staphylococcus aureus* Act as Intracellular Toxins That Kill Infected Osteoblasts

**DOI:** 10.1371/journal.pone.0063176

**Published:** 2013-05-14

**Authors:** Jean-Philippe Rasigade, Sophie Trouillet-Assant, Tristan Ferry, Binh An Diep, Anaïs Sapin, Yannick Lhoste, Jérémy Ranfaing, Cédric Badiou, Yvonne Benito, Michèle Bes, Florence Couzon, Sylvestre Tigaud, Gérard Lina, Jérôme Etienne, François Vandenesch, Frédéric Laurent

**Affiliations:** 1 Institut National de la Santé et de la Recherche Médicale (INSERM) U1111, University of Lyon, Lyon, France; 2 National Reference Center for Staphylococci, Hospices Civils de Lyon, Bron, France; 3 Department of Clinical Microbiology, Northern Hospital Group, Hospices Civils de Lyon, Lyon, France; 4 Division of Infectious Diseases, Department of Medicine, University of California San Francisco, San Francisco, California, United States of America; University of Edinburgh, United Kingdom

## Abstract

Epidemic community-acquired methicillin-resistant *Staphylococcus aureus* (CA-MRSA) is associated with more severe and acute forms of osteomyelitis than healthcare-associated (HA-) MRSA. Although *S. aureus* is now recognized as a facultative intracellular pathogen, the contribution of osteoblast invasion by CA-MRSA to the pathogenesis of osteomyelitis is unknown. Using an ex vivo model of intracellular infection of human osteoblasts, we demonstrated that CA-MRSA strains of diverse lineages share an enhanced ability to kill infected osteoblasts compared to HA-MRSA. Cytotoxicity comparisons of CA-MRSA isogenic deletion mutants revealed that phenol-soluble modulins (PSMs), a class of membrane-damaging exoproteins that are expressed at higher levels in CA-MRSA than in HA-MRSA, are involved in this osteoblast killing, whereas other major CA-MRSA virulence determinants, the Panton-Valentine leukocidin and alpha-toxin, are not involved. Similarly, functional *agr* and *sar*A regulators, which control the expression of PSMs and alpha-toxin, were required for the expression of the intracellular cytotoxic phenotype by CA-MRSA, whereas the *sae*RS regulator, which controls the expression of alpha-toxin but not PSMs, had no impact on cytotoxicity. Finally, PSM transcript levels determined by quantitative reverse-transcriptase PCR were significantly higher in CA-MRSA than in HA-MRSA strains and associated with cell damage in MRSA-infected osteoblasts. These findings provide new insights into the pathogenesis of severe CA-MRSA osteomyelitis and unravel a novel virulence strategy of CA-MRSA, based on the invasion and subsequent killing of osteoblasts by PSMs acting as intracellular toxins.

## Introduction

*Staphylococcus aureus* is the leading cause of osteomyelitis, which is defined as an infection of the bone [1]. This versatile pathogen has evolved a remarkable ability to resist antibiotics such as methicillin and other beta-lactams, complicating the management of osteomyelitis [2]. Until the 1990s, methicillin resistance was recognized as a specific trait of healthcare-associated *S. aureus* (HA-MRSA), which was first described in the early 1960s [3]. The incidence of community-acquired (CA-) MRSA infections has since dramatically increased in several countries [4], and this pandemic has altered the clinical landscape of osteomyelitis, particularly in the pediatric setting [5], [6]. In the United States, CA-MRSA infections are more frequent than their methicillin-susceptible counterparts [7]–[10], and the dissemination of these strains has been coincident with an increase in both the incidence and the severity of osteomyelitis [5], [9]–[12]. Children with osteomyelitis caused by CA-MRSA, compared to other *S. aureus* lineages, exhibit greater systemic inflammatory responses [13], experience longer durations of fever and longer hospital stays [5], [10], and more frequently require surgical procedures [5]. Case series also suggested that these patients often require admission to the intensive care unit [6], [9]. Notably, CA-MRSA infections have added to, rather than replaced, infections caused by other microorganisms, including methicillin-susceptible *S. aureus* (MSSA).

Investigations of the basis of CA-MRSA virulence are crucial for understanding its pathogenesis and the development of novel therapeutics against these recently emerged pathogens. Data from in vitro and animal models have shown that the virulence potential of CA-MRSA is multifactorial. This virulence potential has evolved via the acquisition of the *pvl* genes encoding the Panton-Valentine leukocidin (PVL) and through the increased expression of core genome-encoded toxins, mainly alpha-toxin and phenol-soluble modulins (PSMs) [8]. These pore-forming toxins induce apoptosis and lysis in different cell types. PVL and PSMs target immune effector cells such as neutrophils [8], while alpha-toxin targets a much wider spectrum of cells, including erythrocytes, alveolar epithelial cells [14], endothelial cells [15], lymphocytes, and monocytes [16]. Experimental investigations of CA-MRSA virulence have mainly focused on models of skin and soft tissue infections or pneumonia because these diseases are the most frequent or the most severe, respectively, in the spectrum of CA-MRSA infections [17]. As a consequence, few experimental data are available regarding the pathogenesis of CA-MRSA osteomyelitis. PVL has been shown to contribute to the severity of infection in a rabbit model of osteomyelitis [18]. The expression of the *S. aureus* surface protein A, although not specific to CA-MRSA strains, is also associated with bone destruction through its binding to the tumor necrosis factor receptor 1 of osteoblasts [19]–[21]. However, the roles of CA-MRSA-specific virulence determinants other than PVL are unknown. Direct interactions of *S. aureus* with osteoblasts are crucial in the pathogenesis of osteomyelitis [22], [23]. The ability of *S. aureus* to invade and gain access to the cytoplasm of so-called non-professional phagocytes such as osteoblasts has gained increased attention [22]–[25] and is now regarded as a key factor in therapy-refractive infections [26], [27].

The primary objective of this work was to compare the ability of CA-MRSA and HA-MRSA strains to invade and damage human osteoblasts in an ex vivo model. To achieve adequate representation of MRSA strains circulating worldwide, 35 strains of the major CA-MRSA and HA-MRSA lineages were investigated. Our secondary objective was to determine if specific virulence determinants were associated with either osteoblast invasion or killing by MRSA. The roles of PVL, alpha-toxin, and PSM production and of the regulators *agr*, *sar*A, and *sae*RS in the virulence of MRSA during intracellular infection were examined. Finally, we investigated whether osteoblast killing was associated with the expression levels of the bacterial genes encoding alpha-toxin, PSMs and the *agr* effector RNAIII.

## Results

### Intracellular CA-MRSA causes Higher Osteoblast Damage than HA-MRSA

We examined the cytotoxicity induced in human osteoblasts by 35 genetically diverse clinical strains of MRSA selected from the collection of the French National Reference Center for Staphylococci. These strains belonged to 3 major lineages of *pvl^+^* CA-MRSA, namely sequence type (ST)8, pulsotype USA300, staphylococcal chromosomal cassette *mec* (SCC*mec*) IV (ST8-USA300-IV clone), the ST80-IV European clone, and the ST30-USA1100-IV Southwest Pacific clone [28], and to 4 major lineages of HA-MRSA, namely the ST239-III Brazilian clone, the ST228-I Southern Germany clone, the ST8-EMRSA2-IV Lyon clone, and the ST22-EMRSA15-IV Barnim clone (n = 5 strains each) [29], [30].

The infection protocol was comprised of a 2 h co-culture step of MRSA and MG-63 osteoblastic cells in antibiotic-free medium with a bacteria-host cell ratio of 100, followed by a selection step in medium containing gentamicin and lysostaphin to kill non-internalized bacteria. After 24 h of incubation, the *S. aureus*-induced cytotoxicity was estimated by a lactate dehydrogenase (LDH) assay. The results were reported as the mean and 95% CI of the n-fold change in LDH compared to cells infected with the *S. aureus* reference strain 8325-4 (control); each strain was tested in duplicate.

**Figure 1:**
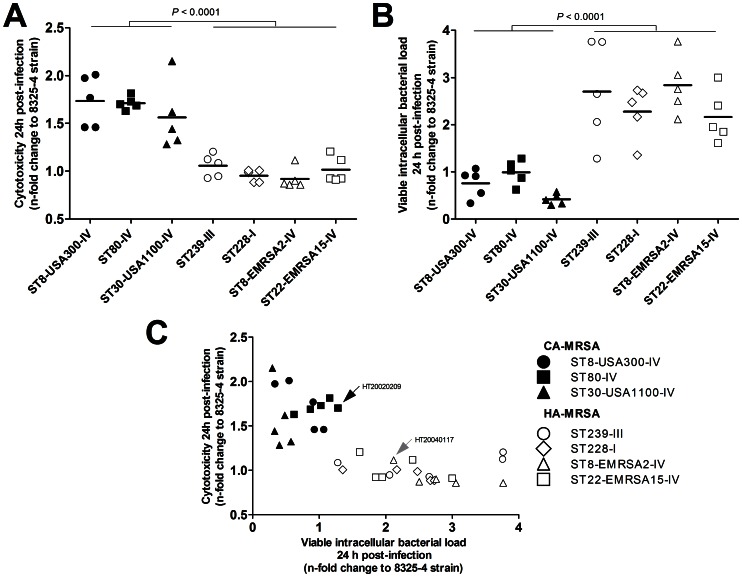
Viable intracellular bacterial loads and host cell damage differentiate CA-MRSA and HA-MRSA strains in a model of intracellular challenge of cultured osteoblasts. Osteoblastic MG-63 cells were infected with one of 35 *S. aureus* strains belonging to 3 distinct CA-MRSA lineages (closed marks) and 4 HA-MRSA lineages (open marks) at an MOI of 100∶1 and incubated for 24 h. Cytotoxicity was estimated by quantifying the LDH release by damaged cells. The infected cells were lysed, and viable intracellular bacterial counts were enumerated. All results were expressed as the n-fold change relative to the *S. aureus* 8325-4 control strain and were derived from duplicate experiments. The *P*-values were calculated using Welch’s *t*-test. (A) Comparison of the relative cytotoxicity of CA-MRSA and HA-MRSA strains. (B) Comparison of the viable intracellular bacterial loads in osteoblasts infected with CA-MRSA and HA-MRSA strains. (C) Plot of relative cytotoxicity and intracellular bacterial loads, indicating differences between the CA-MRSA and HA-MRSA strains. Strains HT20020209 and HT20040117, which were included in the kinetics experiments in Figure 2, are indicated by arrows.

A significant difference was observed in the capacity of CA-MRSA and HA-MRSA to induce osteoblast damage after 24 h. The relative LDH release by CA-MRSA-infected cells was 1.7-fold higher than that by HA-MRSA-infected cells (1.67 [1.53–1.81] vs. 0.99 [0.93–1.04], respectively; *P*<0.0001, Welch’s *t*-test; Figure 1A and Table S1). ANOVA followed by Tukey’s HSD post-hoc test was used to determine if the cytotoxicity was also dependent on the lineage of the strains. Pairwise comparisons showed that (i) any of the 3 CA-MRSA lineages induced significantly higher LDH release than any of the 4 HA-MRSA lineages (*P*<0.01 for all differences) and (ii) no significant difference in LDH release was observed between the lineages within the CA-MRSA or HA-MRSA groups.

### The Intracellular Bacterial Load is Higher in HA-MRSA- than CA-MRSA-infected Osteoblasts

The viable intracellular bacterial loads (VIBL) within MRSA-infected osteoblasts were determined using the infection assay described above, followed by the osmotic lysis of infected cells at 24 h post-infection to release the bacteria, which were enumerated by plate counting. The results were expressed as the mean and 95% CI of the n-fold change in the VIBL compared to cells infected with the *S. aureus* strain 8325-4 (control) and were derived from the same experiments as those used to quantify cytotoxicity.

The relative VIBL was 3.5-fold higher in HA-MRSA-infected osteoblasts than in CA-MRSA-infected osteoblasts (2.50 [2.15–2.84] vs. 0.72 [0.54–0.91], respectively; *P*<0.0001; Fig 1B and Table S1). The differences between the lineages were analyzed using the same ANOVA procedure as described above. Pairwise comparisons showed that (i) the relative VIBL was significantly higher among the 4 HA-MRSA lineages than the 3 CA-MRSA lineages (*P*<0.05 for all differences, Tukey’s HSD test) and (ii) no significant difference in the relative VIBL was observed between the lineages within the CA-MRSA or HA-MRSA groups.

### The Lower Intracellular Bacterial Load of CA-MRSA is not Explained by Host Cell Killing

Following the observation that CA-MRSA induced both higher cytotoxicity and lower VIBL than did HA-MRSA, we tested the hypothesis that cytotoxicity was negatively correlated with VIBL. Because bacteria that kill their host cells are released into the extracellular space and excluded from the intracellular bacterial pool, the higher cytotoxicity of a given strain could directly yield a lower VIBL. We thus searched for an association between cytotoxicity and VIBL with and without controlling for the CA-MRSA or HA-MRSA status of the strain. Figure 1C shows a plot of relative LDH release against VIBL. The VIBL was significantly associated with cytotoxicity levels upon simple regression analysis (*P*<0.0001, *F*-test). However, multiple linear regression controlling for CA-MRSA or HA-MRSA status demonstrated that there was no independent association between VIBL and cytotoxicity (*P* = 0.6).

**Figure 2:**
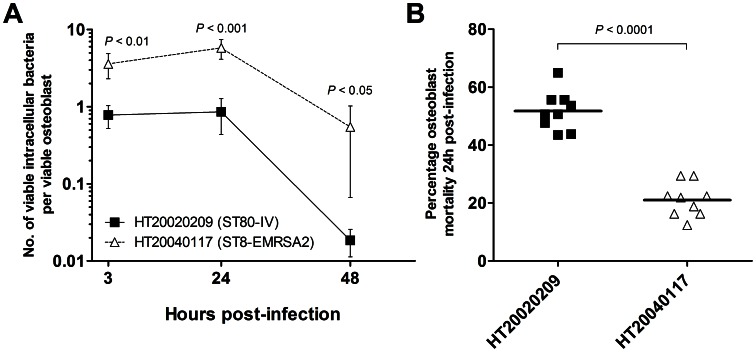
Kinetics of the intracellular passage and survival of representative CA-MRSA and HA-MRSA strains and the mortality of infected osteoblasts. The ST80-IV CA-MRSA strain HT20020209 (closed marks) and the ST8-EMRSA2-IV HA-MRSA strain HT20040117 (open marks) were used to inoculate MG-63 osteoblastic cells. The indicated *P*-values were calculated using Welch’s *t*-test, and the results were derived from three independent experiments in triplicate. (A) Kinetics experiments of intracellular bacterial passage and survival. At each time point, the viable intracellular bacteria and osteoblasts were quantified to calculate the no. of viable bacteria per osteoblast. The results are shown as the means ±95% CI. (B) The percent mortality of infected osteoblasts 24 h post-infection confirms the strong cytotoxic effect of ST80-IV *S. aureus* compared to ST8-EMRSA2-IV.

To further explore the relationships between bacterial invasion, intracellular persistence, and the CA-MRSA or HA-MRSA status of the strains, we quantified the number of viable bacteria per viable osteoblast in kinetics experiments. The first time point was 3 h after the beginning of the infection step to reflect the efficiency of the invasion process. Subsequent time points were taken at 24 and 48 h after infection to investigate the clearance of intracellular bacteria with respect to the initial VIBL. Two strains (the ST80-IV CA-MRSA strain HT20020209 and the ST8-EMRSA2-IV HA-MRSA strain HT20040117) were randomly selected from the 35 MRSA strains and included in these experiments (see arrows in Figure 1C). The results are reported as the means and 95% CI derived from three independent experiments in triplicate. At 3 h post-infection, the osteoblasts harbored an average of 0.77 [0.52–1.03] ST80-IV cells and 3.59 [2.30–4.89] ST8-EMRSA2-IV cells, which corresponded to approximate intracellular passages of 1% and 4%, respectively, of the bacterial inoculum set at 100 bacteria per osteoblast (Figure 2A). These figures remained stable from 3 to 24 h post-infection, at which time the bacteria per osteoblast ratios were 0.86 [0.44–1.27] and 5.78 [4.13–7.44] for the ST80-IV and ST8-EMRSA2-IV strains, respectively. Significant bacterial clearance occurred between 24 and 48 h, at which time the ratios fell to 0.02 [0.01–0.03] for the ST80-IV strain and 0.55 [0.06–1.03] for the ST8-EMRSA2-IV strain, corresponding to 46.3- and 10.6-fold reductions, respectively, in the bacterial load. Comparisons of the ST8-EMRSA2-IV and ST80-IV strains revealed that the bacteria per osteoblast ratios after 3, 24, and 48 h of incubation were 4.6-, 6.8-, and 29.5-fold higher, respectively, for the HA-MRSA strain than for the CA-MRSA strain (*P*<0.05 for all differences, Welch’s *t*-test). Collectively, these findings indicated that the invasion process itself and the ability to survive intracellularly after invasion were less efficient in the CA-MRSA strain HT20020209 than in the HA-MRSA strain. Moreover, these experiments confirmed that the difference in the amounts of intracellular bacteria between CA-MRSA and HA-MRSA was independent of the host cell death caused by CA-MRSA.

Additional experiments to investigate osteoblast infection were conducted as described above using the same two strains, HT20020209 and HT20040117, to estimate the mortality of infected osteoblasts. The results were reported as the means and 95% CI derived from three independent experiments in triplicate. The percent mortality in osteoblasts infected with the CA-MRSA strain HT20020209 and the HA-MRSA strain HT20040117 were 51.8% [46.6–56.9] and 21.0% [16.6–25.5], respectively (*P*<0.0001, Welch’s *t*-test; Figure 2B). These results, together with those of the infection kinetics experiments, confirmed the potent cytotoxic activity of intracellular CA-MRSA by showing that an average intracellular load of one bacterium per host cell resulted in the death of half of the host cell population by 24 h.

### PVL is not Involved in the Intracellular Virulence of CA-MRSA

**Figure 3:**
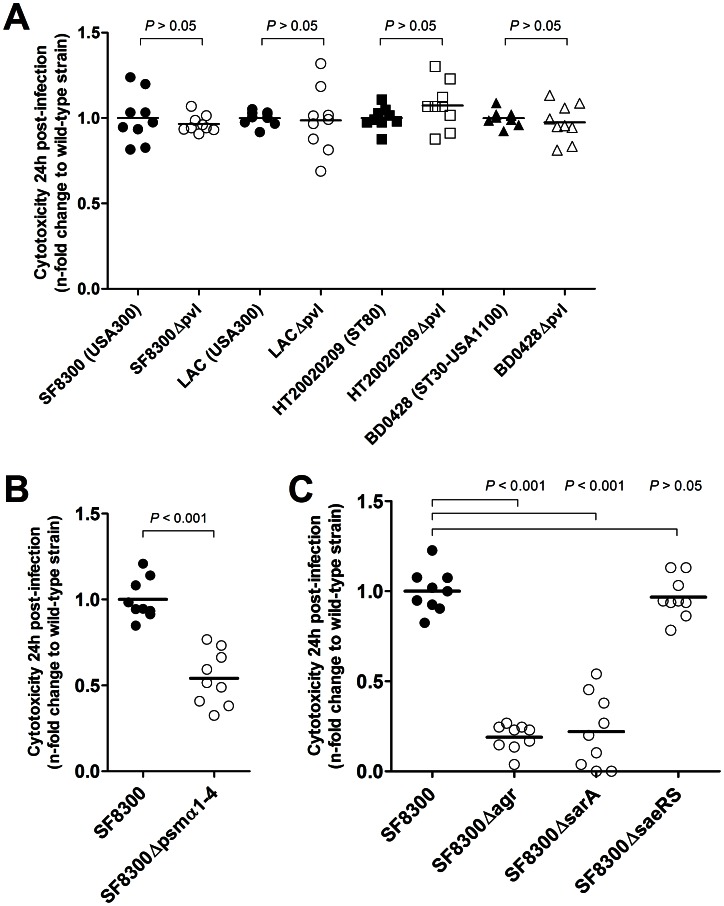
Impact of PVL, alpha-type PSMs and the major regulatory systems *agr*, *sar*A, and *sae* RS on the cytotoxicity of CA-MRSA toward osteoblasts. In all experiments, cytotoxicity was estimated by quantifying LDH release by CA-MRSA-infected MG-63 osteoblastic cells at 24 h post-infection. The results were derived from three independent experiments performed in triplicate and expressed as the n-fold change in LDH release of each isogenic deletion mutant (open marks) relative to the wild-type strain (closed marks). All *P*-values were calculated using Welch’s *t*-test. (A) Effect of the inactivation of *pvl* genes on the cytotoxicity of genetically diverse CA-MRSA. No significant differences were observed between the cytotoxicity of the wild-type and Δ*pvl* strains, indicating that PVL is not involved in CA-MRSA-induced cytotoxicity toward osteoblasts. (B) Impact of the inactivation of the *psmα*1-4 genes on the cytotoxicity of the USA300 CA-MRSA strain SF8300. The strain SF8300Δ*psmα*1-4 was significantly less cytotoxic than the wild-type strain, thus indicating that alpha-type PSMs are involved in the cytotoxic phenotype. (C) Impact of the inactivation of the *agr*, *sar*A, and *sae*RS genes on the cytotoxicity of strain SF8300. Strains SF8300Δ*agr* and SF8300Δ*sar*A, but not SF8300Δ*sae*RS, were significantly less cytotoxic than the wild-type strain.

Although PVL specifically targets immune cells, this toxin has been shown to bind mitochondria and to cause Bax-independent apoptosis through the mitochondrial pathway [31]. Hence, direct delivery of PVL by intracellular CA-MRSA in the cytoplasm of infected osteoblasts may allow the toxin to gain access to the mitochondria without the need for immune cell type-specific binding to the plasma membrane. PVL is found in most CA-MRSA but not HA-MRSA strains and is expressed at toxic levels as long as the corresponding genes are present in the genome [32], [33]. Therefore, we used a loss-of-function approach to examine the influence of PVL on cytotoxicity by using isogenic *pvl* ^+/−^ strains belonging to the three CA-MRSA lineages investigated in the previous experiments. With respect to ST8-USA300-IV, strains LAC and SF8300, as well as their Δ*pvl* derivatives LACΔ*pvl* and SF8300Δ*pvl*, have been described previously [34], [35]. The following mutants were constructed by allelic replacement: the LUG1800 Δ*pvl* mutant of the ST80-IV strain HT20020209, and the BD0448 Δ*pvl* mutant of the ST30-USA1100-IV strain BD0428. The cytotoxicity toward osteoblasts was assessed after 24 h of infection using the same procedure as described above. The results of three experiments performed in triplicate are presented in Figure 3A. No significant differences in cytotoxicity were observed between the wild-type and Δ*pvl* strains in the three lineages investigated (*P*>0.05 for all comparisons, Welch’s *t*-test), thus eliminating a potential role for PVL in the increased cytotoxicity of CA-MRSA toward osteoblasts.

### Alpha-toxin Production Level is not Correlated with Osteoblast Damage

**Figure 4:**
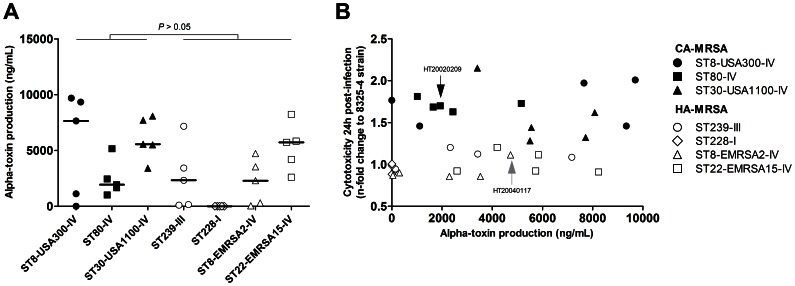
Comparison of alpha-toxin production in CA-MRSA and HA-MRSA strains. Alpha-toxin production in 24-h bacterial culture supernatants was quantified by sandwich ELISA. (A) Alpha-toxin production was not significantly different in the CA-MRSA and HA-MRSA strains, although strong variations were observed between different lineages and within the strains of a given lineage. Horizontal lines represent median values. The *P*-value was calculated using a non-parametric Mann-Whitney *U*-test. (B) Plot of MRSA cytotoxicity toward osteoblasts against alpha-toxin production. Note that one ST8-USA300-IV CA-MRSA strain had no measurable alpha-toxin production but was more cytotoxic than any of the HA-MRSA strains. Strains HT20020209 and HT20040117, which were included in the kinetics experiments of Figure 2, are indicated by arrows.

The *hla* gene encoding alpha-toxin belongs to the core genome of *S. aureus*, and the expression level of this toxin has been shown to affect strain-specific virulence [36]. We thus searched for an association between alpha-toxin production and cytotoxicity. The in vitro production of alpha-toxin by MRSA strains and by the *S. aureus* strain 8325-4 was quantified in duplicate using a sandwich ELISA and reported as ng/mL. Because the data were not normally distributed upon visual inspection, we used non-parametric tests for the statistical analysis and reported the medians and interquartile ranges (IQR) instead of means and the 95% CI. Alpha-toxin production tended to be higher in CA-MRSA than in HA-MRSA strains, but this difference did not reach statistical significance (median and IQR, 5153 ng/mL [1790-7683] vs. 2310 ng/mL [36–4326], respectively; *P* = 0.074, two-tailed Mann-Whitney *U*-test; Figure 4A and Table S1). Among the 35 MRSA strains investigated, 7 strains produced low amounts of alpha-toxin (<50 ng/mL), including the 5 ST228-I HA-MRSA strains (100%), 1 ST8-EMRSA2-IV HA-MRSA strain (20%), and unexpectedly, 1 ST8-USA300-IV CA-MRSA strain (20%). We plotted the relative cytotoxicity of the MRSA strains against the alpha-toxin activity (Figure 4B) and searched for an association between these factors using a non-parametric correlation analysis. A moderate rank correlation was found (Spearman’s coefficient = 0.31) that did not reach statistical significance (*P* = 0.069). No association was found by multiple linear regression analysis controlling for the CA-MRSA or HA-MRSA status (*P* = 0.75, *F*-test). Notably, the 8325-4 control strain, which had the highest alpha-toxin production (28.8 µg/mL) due to a previously described chromosomal defect [37], was less cytotoxic toward osteoblasts than any of the CA-MRSA strains, including the alpha-toxin-deficient USA300 strain. Conversely, the alpha-toxin-deficient ST8-USA300-IV CA-MRSA strain was still more cytotoxic than any HA-MRSA strain. Collectively, these findings do not support a role of alpha-toxin in the increased cytotoxicity of CA-MRSA toward osteoblasts in our model.

### Alpha-type PSMs are Required for Intracellular Virulence

PSMs are core genome-encoded amphipathic peptides that have been associated with CA-MRSA virulence in animal models [38]–[40] and are able to recruit, activate, and lyse neutrophils [38], [41]. Several PSMs are present in *S. aureus* genome, mainly alpha- and beta-type PSMs and the delta-toxin. Among the PSM family members, alpha-type PSMs are the most strongly associated with neutrophil activation and virulence in animal model [38]. The cytotoxic phenotypes of the previously described CA-MRSA strain SF8300 and of its isogenic derivative SF8300Δ*psm*α1-4, which lacks alpha-type PSMs, were compared [42] (Figure 3B). The inactivation of the alpha-type PSMs induced a significant decrease in osteoblast damage after 24 h of incubation, indicating that the expression of alpha-type PSMs by CA-MRSA is associated with a cytotoxic phenotype.

### PSM-controlling Regulators *agr*A and *sar*A, but not *sae*RS, are Required for Intracellular Virulence

Toxin expression in *S. aureus* is tightly controlled by a regulatory network involving several regulators, including *agr*, *sar*A, and *sae*RS [36]. All of these three regulators are required for alpha-toxin expression, while only *agr*A and *sar*A impact PSM expression [38], [43]. The increased toxin expression and virulence of CA-MRSA strains has been attributed to the increased expression of these systems [36], [44]. We thus investigated the respective contributions of each of these 3 regulators to cytotoxicity by constructing isogenic derivatives of strain SF8300 that lack *agr*A, *sar*A, or *sae*RS. Both the SF8300Δ*agr* and SF8300Δ*sar*A strains but not the SF8300Δ*sae*RS strain induced significantly less damage in infected osteoblasts than the wild-type SF8300 strain (Figure 3C). These results indicate that the virulence determinants responsible for osteoblast death after invasion are under the control of *agr*A and *sar*A but not *sae*RS, which is consistent with a major role of PSMs in intracellular virulence.

### In vitro Transcript Levels of *psm*α, but not *hla* nor RNAIII, are Associated with Cytotoxicity

**Figure 5:**
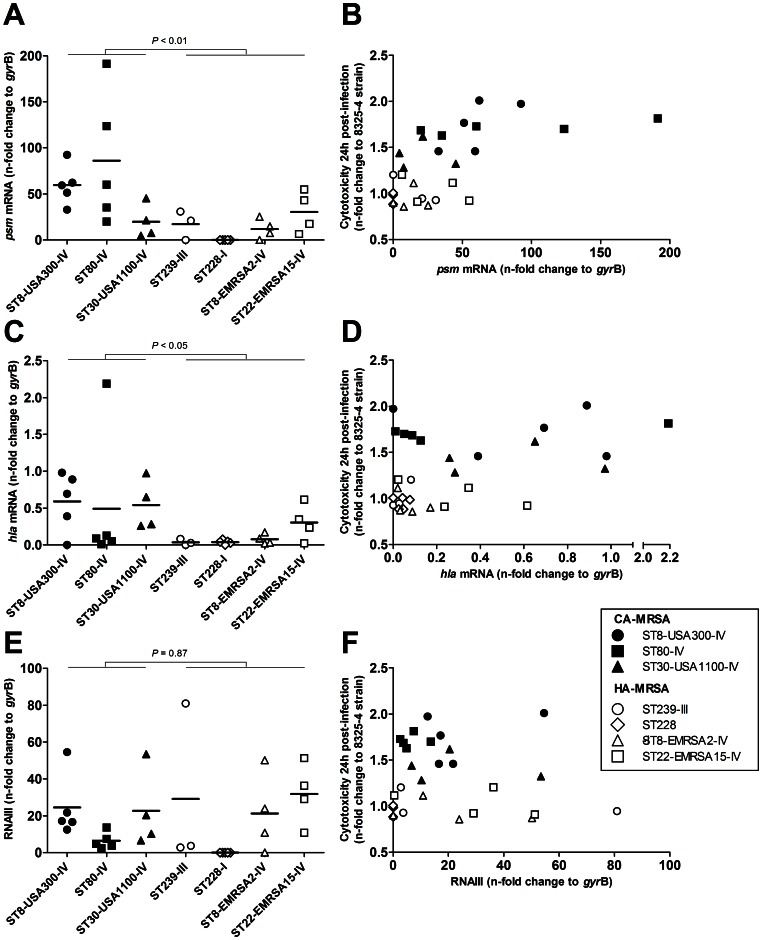
Transcript levels of *psmα*, but not *hla* nor RNAIII, are associated with cytotoxicity in MRSA. Relative transcript levels of *psmα*, *hla* and RNAIII were determined using quantitative reverse-transcriptase PCR and expressed as n-fold change to the internal *gyr*B standard. (A) *psmα* transcript levels were globally higher in CA-MRSA than in HA-MRSA, and (B) were significantly associated with cytotoxicity in simple linear regression analysis (*P*<0.001, *F*-test) and in multivariate analysis controlling for the CA-MRSA or HA-MRSA status of the strain (*P*<0.0001). (C) *hla* transcript levels were higher in CA-MRSA than in HA-MRSA and (D) were associated with cytotoxicity in univariate analysis, but not in multivariate analysis. (E) RNAIII transcripts levels were strain- and lineage-dependant but showed no global difference between CA-MRSA and HA-MRSA; moreover (F) they were not associated with cytotoxicity.

To further investigate the correlation of PSMs and alpha-toxin expression with intracellular virulence, we quantified the transcript levels of *psm*α, *hla* and the *agr* effector RNAIII using relative quantitative reverse-transcription PCR as described elsewhere with modifications [40]. Transcripts levels were expressed as n-fold change to the *gyr*B internal standard and reported as the mean and 95% CI. The 35 clinical MRSA strains were included in these experiments, with the exception of 5 strains in which the RNA yield after extraction was consistently insufficient (ST239-III, n = 2, and ST30-USA1100-IV, ST8-EMRSA2-IV and ST22-EMRA15-IV, n = 1 each). Strains SF8300, SF8300Δ*agr*, SF8300Δ*sar*A and SF8300Δ*sae*RS were also included. The relative levels of *psm*α and *hla* transcripts were both globally higher in CA-MRSA as compared to HA-MRSA (57.7 [31.3–84.0] vs. 13.9 [5.4–22.3], *P*<0.01, Welch’s *t*-test, and 0.54 [0.23–0.85] vs. 0.11 [0.04–0.19], *P*<0.05, respectively; Figure 5A and 5C). The levels of RNAIII transcripts were mostly strain- and lineage-dependent and showed no global difference between CA-MRSA and HA-MRSA (17.5 [8.84–26.2] vs. 18.7 [6.69–30.78], *P* = 0.87; Figure 5E). Of note, the 5 ST228-I HA-MRSA strains were *agr*-defective. In univariate analysis, the relative cytotoxicity was strongly associated with the *psm*α transcript level (*P*<0.001) and, to a lesser extent, to the *hla* transcript level (*P*<0.05), but not to the RNAIII transcript level. A strong rank correlation was found between the ARNIII transcript levels and both *psm*α (*P*<0.001) and *hla* (*P*<0.01) transcript levels but these associations were not significant in linear regression. In multivariate analysis, the *psm*α transcript levels and the CA-MRSA or HA-MRSA group were independently associated with cytotoxicity (*P*<0.05 for both regression coefficients), but not the *hla* (*P* = 0.80) nor the RNAIII (*P* = 0.67) transcript levels. As expected, transcripts of *hla* were undetectable in strains SF8300Δ*agr*, SF8300Δ*sar*A and SF8300Δ*sae*RS. Transcripts of both *psm*α and RNAIII were undetectable in strain SF8300Δ*agr* but were found at levels comparable to those of the wild-type strain in strains SF8300Δ*sar*A and SF8300Δ*sae*RS. These results are consistent with the recent report that the *sae*RS regulator has no significant impact on PSM expression, and that the *sar*A regulator does not regulate PSM transcription but mainly reduces the post-secretion degradation of PSMs by downregulating the expression of the aureolysin protease [43].

## Discussion

The emergence of CA-MRSA as a cause of osteomyelitis has been associated with an increase in both the incidence and severity of this disease. A better understanding of the virulence mechanisms of CA-MRSA in osteomyelitis may help improve management strategies and establish targeted therapies. Our results indicate that the invasion of osteoblasts by CA-MRSA and the intracellular expression of *psm*α by such strains results in extensive cell damage. This potential virulence trait might contribute to the increased severity of osteomyelitis caused by CA-MRSA relative to that caused by canonical MRSA strains.

The invasion of osteoblasts by *S. aureus* has been extensively studied over the past decade, and interpretations of the clinical significance of this phenomenon have exclusively focused on chronic and indolent forms of osteomyelitis [22], [23]. More specifically, the intracellular passage of bacteria has been considered a means by which *S. aureus* protects itself, escapes antibiotics and the immune response of the host, and establishes a latent bacterial reservoir that is potentially responsible for chronicity and recurrence [26], [27]. In accordance with this interpretation, our investigations of the intracellular survival of HA-MRSA strains demonstrated the ability of these strains to persist within osteoblasts without causing extensive damage. Conversely, this interpretation of osteoblast invasion as an underlying mechanism for chronicity and indolence does not appear relevant to severe and acute CA-MRSA osteomyelitis. Indeed, our data indicate that an intracellular bacterial load of one CA-MRSA cell per osteoblast is sufficient to induce the death of half of the osteoblast population within 24 h (Figure 2). Hence, given the poor ability of the CA-MRSA strains to persist intracellularly and the extensive damage caused to infected host cells, it is more likely that osteoblast invasion by CA-MRSA is part of a pathogenesis strategy based on aggression and damage rather than self-protection, a view that is consistent with previous clinical observations [5], [6], [9], [10], [13].

Alpha-toxin has previously been shown to induce apoptosis in endothelial cells infected with *S. aureus* [45] and to contribute to CA-MRSA pathogenesis in a murine model of pneumonia [46]. Alpha-toxin production and transcription tended to be higher in CA-MRSA than in HA-MRSA strains in our experiments, but several lines of evidence indicate that this toxin was not responsible for the death of the infected osteoblasts. First, we failed to demonstrate any association of alpha-toxin production with cytotoxicity; second, cytotoxicity was conserved in both the non-hemolytic USA300 strain and its hemolytic counterparts; and third, the 8325-4 reference strain, which had the highest alpha-toxin production among our strain collection, was less cytotoxic than any of the 15 CA-MRSA strains. Finally, the inactivation of *sae*RS in strain SF8300, which resulted in an alpha-toxin-deficient phenotype confirmed by undetectable *hla* transcript levels in quantitative reverse-transcription PCR assays, had no impact on cytotoxicity.

The contribution of PSMs to CA-MRSA virulence was first described in a murine model of skin and soft tissue infection [38]. The PSM family is comprised of several proteins: the δ-toxin, α-type PSMs 1-4, β-type PSMs 1-2, and the SCC*mec*-encoded PSM-*mec* (reviewed in [47]). Among these proteins, alpha-type PSMs were shown to be able to recruit, activate, and lyse neutrophils [38], thus exhibiting a role in pathogenesis that appeared similar to that of PVL. Whereas neutrophil chemotaxis and activation by PSMs occur at nanomolar concentrations and involve PSM detection by the neutrophil formyl peptide receptor 2 (FPR2) in vitro [48], neutrophil lysis requires micromolar concentrations of alpha-type PSMs, is receptor-independent [38], [47], [48], and is thought to involve lipid membrane disruption caused by the amphipathic alpha-helix structure of PSMs [38]. Interestingly, it has recently been shown that human serum components inhibit both the FPR2-activating and neutrophil lysis properties of PSMs, casting doubt on the relevance of PSMs as extracellular toxins [49]. Our findings that PSMs act as intracellular toxins are thus in line with the aforementioned observations. Indeed, *S. aureus* cells that invade non-professional phagocytes such as osteoblasts initially remain trapped in phagosomes [50]. It is thus likely that a sustained expression of PSMs in this confined environment allows these toxins to accumulate. However, the exact mechanism by which PSMs contribute to the death of the host cell are unknown. Of note, the overexpression of alpha-type PSMs by *S. aureus* is not associated with phagosomal escape [51]. Such escape, or at least the permeabilization of the phagosome membrane, has been shown to involve other factors such as delta-toxin. However, delta-toxin-mediated phagosome membrane disruption requires the presence of a functional beta-toxin [51], while it is well-known that most *S. aureus* clinical strains, including CA-MRSA, harbor a non-functional beta-toxin because of the insertion of various phages in the beta-toxin-encoding gene *hlb* [28], [52]. In this context, some authors have hypothesized that phagosomal escape in CA-MRSA might involve other delta-toxin co-factors that are still to be determined or, alternatively, that *S. aureus* exposure to reactive oxygen species in the phagosomal environment might induce the excision of the beta-toxin-converting phages, thus allowing a functional beta-toxin expression [53].

Using Δ*agr*, Δ*sar*A, and Δ*sae*RS mutants of the CA-MRSA strain SF8300, we demonstrated that only the first two regulators are related to the intracellular cytotoxic phenotype of CA-MRSA. These findings correlate with the major role of PSMs in this phenotype: (i) PSM secretion by *S. aureus* is under direct control of *agr* [38]; (ii) *sar*A reduces the post-secretion degradation of PSMs by downregulating the expression of the aureolysin (*aur*) protease and, to a lesser extent, regulates PSM secretion by upregulating *agr* [43]; and (iii) *sae*RS expression has no significant impact on PSM expression [43].

Previous research on the basis of CA-MRSA virulence in the specific context of osteomyelitis has understandably focused on the role of PVL. Cremieux et al. used a rabbit model of osteomyelitis to demonstrate that PVL contributes to the severity of infection in terms of bone deformation, extra-osseous involvement, and the systemic inflammatory response [18], in keeping with clinical observations in human [10], [54]. These outcomes are most likely related to the potent pro-inflammatory properties of PVL, including the capacity of PVL to recruit, activate, and lyse immune cells at the site of infection. However, recent CA-MRSA research has emphasized the remarkably complex virulence mechanisms of these pathogens, as well as the risk of oversimplifying CA-MRSA virulence by considering only the individual action of a single bacterial factor [17]. The multiplicity and frequent functional redundancy of CA-MRSA virulence determinants are major obstacles to our understanding of CA-MRSA virulence [8], and a decade of intensive research has been necessary to outline an integrated view of the relative contributions of PVL and alpha-toxin to CA-MRSA pathogenesis [17]. In this context, our observation that CA-MRSA strains of several genetically distinct lineages share an enhanced ability to kill osteoblasts after intracellular passage through a PSM-dependent mechanism adds to our knowledge of the potential pathogenesis strategies of CA-MRSA. Put together, PSM-related killing of CA-MRSA-infected osteoblasts and PVL-related recruitment and lysis of immune cells sketch the outlines of a new model for CA-MRSA pathogenesis in osteomyelitis, in which concomitant intracellular and extracellular activity of this pathogen both contribute to local tissue damage. The relative contributions of indirect PVL-related tissue damage and of PSM-related post-invasion osteoblast killing in the clinical course of CA-MRSA osteomyelitis remain to be determined. To address this question, future studies should focus on animal models of osteomyelitis involving Δ*pvl*, Δ*psm* and Δ*pvl*-*psm* CA-MRSA strains, and clinical investigations should examine potential correlations between the severity and acuteness of *S. aureus*-induced osteomyelitis and the strain-specific expression level of PSM.

## Materials and Methods

### Bacterial Strains and Growth Conditions

*S. aureus* strain 8325-4 was used as a reference in all experiments [55]. The strain collection of the French National Reference Center for Staphylococci (FNRCS) was searched for CA-MRSA and HA-MRSA isolates representative of prevalent CA-MRSA and HA-MRSA genotypes in Europe according to recent epidemiological data [4], [28], [30]. Five strains of each genotype were selected. The inclusion criteria were based on the molecular characteristics of each strain, as available in the FNRCS database, including the sequence type, SCC*mec* type, and the presence of the *pvl* and *ent*A genes. Genotype-specific inclusion criteria were as follows: ST8, SCC*mec* IV, *pvl* ^+^ for the ST8-USA300-IV clone; ST80, SCC*mec* IV, *pvl* ^+^ for the ST80-IV European clone; ST30, SCC*mec* IV, *pvl* ^+^ for the ST30-USA1100-IV Southwest Pacific clone; ST239, SCC*mec* III for the ST239-III Brazilian clone; ST228, SCC*mec* I for the ST228-I Southern German clone; ST8, SCC*mec* IV, *ent*A^+^ for the ST8-EMRSA2-IV Lyon clone [29]; and ST22, SCC*mec* IV for the ST22-EMRSA15-IV Barnim clone. The lineage of each strain was subsequently confirmed using DNA microarray-based assignment (data not shown) [56]. Of note, DNA microarray results demonstrated that all strains but the 8325-4 strain harbored a non-functional, phage-converted beta-toxin-encoding gene. Clinical data were not considered in the strain selection process because the focus of the present study was the strain genotype; in addition, the number of MRSA strains in the FNRCS collection that had been isolated from documented osteomyelitis cases was too low to restrict the inclusion to such strains.

The strains were stored at −20°C in cryotubes. For each experiment, the strains were first cultivated on Columbia agar supplemented with sheep blood at 37°C for 24 h after thawing. One colony was then used to inoculate brain-heart infusion (BHI) broth. In cell culture infection experiments, the BHI broth was incubated overnight at 37°C, then diluted 5-fold in fresh BHI and further incubated with gyratory shaking for 3 h until mid-exponential phase was reached. Exponential phase cultures were preferred to stationary phase cultures because bacterial adhesins involved in host cell invasion are upregulated in the former [57].

### Construction of Allelic Replacement CA-MRSA Mutants

The *pvl* genes *(lukS-*PV and *lukF-*PV) in the ST80-IV CA-MRSA strain HT20020209 were inactivated by allelic replacement. The Δ*pvl::tetM* mutant LUG1800 was obtained by using pMAD, a thermo-sensitive plasmid containing a constitutively expressed ß-galactosidase gene, which allows the positive selection of double crossing over by detecting ß-galactosidase activity on X*gal* agar plates [58]. A 2.9-kb DNA fragment corresponding to the tetracycline resistance gene *tetM* [59] was cloned into pMAD between two DNA fragments generated by PCR (486 bp and 541 bp) that correspond respectively to the chromosomal DNA regions upstream of *lukS-*PV (up to the start codon) and downstream of *lukF-*PV (from codon 200 to the end). The resulting plasmid, pLUG934, conferred resistance to ampicillin and erythromycin and contained the *lac*Z gene. pLUG934 was electroporated into the *S. aureus* strain RN4220. As the plasmid from RN4220 could not be electroporated into HT20020209, transformation was achieved with phage Φ11 by lysogenizing RN4220/pLUG934 and transfecting HT20020209. The transformants were grown at a non-permissive temperature (37°C) in the presence of 1.5 µg/mL erythromycin to select cells in which the plasmid had been integrated into the chromosome by homologous recombination. To favor the second recombination event, a single colony was grown at 30°C for 10 generations and plated at 37°C overnight. Cells that had lost the plasmid vector through a double cross-over event were detected on X*gal* agar plates. PCR amplification was used to confirm the loss of the *pvl* genes, which were replaced by the *tet*M gene in strain LUG1800.

The *pvl* genes in the ST30-USA1100-IV CA-MRSA strain BD0428 and the *hla*, *psmα*1-4, *agr*A, *sar*A, and *sae*RS genes in the ST8-USA300 CA-MRSA strain SF8300 were inactivated as described previously for the LAC Δ*pvl::spc* strain [34] by allelic replacement of the gene(s) of interest with a spectinomycin resistance cassette.

### Cell Culture

All cell culture reagents were purchased from GIBCO (Paisley, UK). The human osteoblastic cell line MG-63 was purchased from LGC Standards (Teddington, UK) and grown in Dulbecco’s modified Eagle’s medium (DMEM) containing 2 mM L-glutamine, 25 mM HEPES, 10% fetal bovine serum (FBS) and 100 U/mL penicillin and streptomycin (culture medium) at 37°C and 5% CO_2_. The cells were subcultured twice a week and used up to passage 10 after thawing.

### MG-63 Cell Invasion Assay

The intracellular infection of MG-63 cells was performed as described elsewhere with modifications [60]. MG-63 cells were seeded at 50,000 cells/well in 24-well plates and incubated at 37°C with 5% CO_2_ for 48 h in culture medium. Suspensions of mid-exponential phase bacterial cultures were washed, sonicated to minimize clumping, and resuspended in antibiotic-free culture medium at a concentration corresponding to an MOI of 100. The bacterial concentration was normalized using clone-specific regression formulas correlating bacterial density (CFU/mL) with OD at 600 nm, which were established in preliminary experiments. The MOIs were subsequently confirmed by plating the suspensions on agar and counting the bacterial colonies. The MG-63 cells were washed twice in DMEM to remove antibiotics, and normalized bacterial suspensions were added to the wells. The infected cultures were incubated for 30 min at 4°C to allow the bacteria to sediment while blocking internalization, and all of the cultures were simultaneously transferred to 37°C to synchronize the beginning of the internalization step. After a 2 h incubation, the infected cells were washed and further incubated for 1 h in culture medium containing 200 mg/L gentamicin and 10 mg/L lysostaphin to rapidly kill extracellular but not intracellular bacteria. Several strains exhibited decreased susceptibility to gentamicin or lysostaphin when used individually (data not shown), and thus the use of a gentamicin/lysostaphin combination ensured a constant bactericidal activity as verified in preliminary experiments by controlling the sterility of culture supernatants (data not shown). In experiments with time points of 24 and 48 h, the cultures were further incubated for the indicated time in medium containing 40 mg/L gentamicin and 10 mg/L lysostaphin. These lower concentrations resulted in the killing of bacteria cells released upon host cell lysis, thus preventing these bacteria from infecting new host cells. Infected cells that enter apoptosis or necrosis undergo membrane leakage, resulting in the release of the cytosolic enzyme LDH into the culture supernatant, where it can be quantified. At each indicated time point, the cell culture supernatant was removed, and the LDH activity was assessed using a colorimetric method with a Dimension Vista automated clinical chemistry analyzer (Siemens Healthcare Diagnostics, Tarrytown, NY). Cell monolayers were washed to remove antibiotics, lysed by osmotic shock in pure sterile water, and extensively pipetted to achieve the full release of the internalized bacteria. Cell lysates were then sonicated to minimize clumping of the bacteria and spiral-plated in duplicate on agar using a WASP automated plater (AES Chemunex, Bruz, France). After 24 h of incubation, the colonies were enumerated using an EasyCount automated plate reader (AES Chemunex). Due to the large number of experiments required to compare the different MRSA lineages and isogenic MRSA strains, the LDH release and intracellular bacterial counts were expressed relative to the results of the 8325-4 reference strain in experiments involving clinical strains or relative to the respective wild-type strain of each isogenic mutant in experiments involving gene inactivation to control for inter-experiment variations. Conversely, experiments investigating intracellular bacterial survival kinetics, as well as those investigating osteoblast mortality using two representative isolates of ST80-IV (HT20020209) and ST8-EMRSA2-IV (HT20040117), were conducted using three consecutive passages of MG-63 cells. The inter-experiment variation was negligible in these experiments, and thus data normalization was not required. To estimate the number of viable bacteria per viable osteoblast at each time point, the number of viable osteoblasts was quantified microscopically using Trypan blue exclusion, and the numbers of viable intracellular bacteria were quantified as mentioned above. To estimate the percent mortality of osteoblasts 24 h post-infection, LDH release into the supernatant of infected cells was compared to that of uninfected cells that were either left intact (lower control) or fully lysed by osmotic shock (higher control). The percent mortality was calculated as follows: (LDH infected cells - LDH lower control)/(LDH higher control - LDH lower control).

### Alpha-toxin Quantification

Alpha-toxin production by the CA-MRSA and HA-MRSA strains was assessed by means of a sandwich ELISA. In brief, the wells of microtiter plates were coated with an anti-alpha-toxin murine monoclonal antibody (kindly provided by GSK Biologicals) in PBS overnight at room temperature. The unbound monoclonal antibody was washed out twice with a blocking solution of PBS-Tween (0.05%) and milk (5 g/L), followed by incubation with the blocking solution for 1 h at room temperature. Standard dilutions of recombinant alpha-toxin and 24 h bacterial culture supernatant were added to duplicate wells, incubated for 1 h at 37°C, and washed three times, followed by the addition of a rabbit polyclonal anti-alpha-toxin antibody (GSK Biologicals). The microplates were incubated for 1 h at 37°C and washed. Subsequently, a horseradish peroxidase-conjugated swine anti-rabbit polyclonal antibody (DAKO SAS, Trappes, France) was added. The microplates were incubated for 1 h at 37°C and washed before the addition of the tetramethylbenzidine substrate (Sigma Aldrich). The reaction was stopped with H_2_SO_4_ after 30 min, and the plates were read at 450 nm in a microplate reader (Model 680, Bio-Rad). The sandwich ELISA has a lower limit of detection of 2 ng/mL.

### Relative Quantitative Reverse-transcription PCR

Overnight bacterial cultures were used to inoculate a BHI broth and incubated at 37°C with gyratory shaking at 200 rpm. Bacteria were harvested after 10 h to reach the post-exponential growth phase in which the *agr* regulator is activated, in order to approximate the *agr*-activated state of bacteria entrapped in phagosomes [61]. Culture aliquots of 1 mL were centrifuged at 13,000 g, and the pellets were washed with 1 mL of 10 mM Tris buffer and adjusted to an optical density at 600 nm (OD600) of 1, corresponding to approximately 10^9^ *S. aureus* cells/mL. One mL of adjusted and washed bacterial suspension was centrifuged at 13,000 g, and the pellets were treated with lysostaphin (Sigma-Aldrich) at a final concentration of 200 mg/L. The total RNA of the pellets was then purified using the RNeasy Plus Mini Kit (Qiagen) according to the manufacturer’s instructions. The RNA yield was assessed by UV absorbance, and 1 µg of total RNA was reverse transcribed using the Reverse Transcription System (Promega) with random primers, as recommended by the provider. The resulting cDNA was used as the template for real-time amplification of *gyr*B, *psm*α, RNAIII and *hla* using previously published primers [40]. The relative amounts of the *psm*α, RNAIII and *hla* amplicons were determined by quantitative PCR and expressed as fold-change to the *gyr*B internal standard as described elsewhere [62].

### Statistical Analysis

The normality of the data was assessed by visually inspecting the distributions. Two-group differences were analyzed using a two-tailed Welch’s *t-*test for normal data with sufficient sample size (n ≥9) or a two-tailed Mann-Whitney *U*-test for either non-normal data or data with small sample size (n<9). Multiple pairwise comparisons of the means were performed using ANOVA with Tukey’s HSD post-hoc test. Associations of numeric response variables with either numeric or categorical input variables were performed by linear regression. The significance of the regression coefficients was analyzed using Fisher’s *F*-test. Simple linear regression was performed first. Multiple linear regression was then used to control for the CA-MRSA or HA-MRSA status of the strains using a one-step forward selection procedure in which the CA-MRSA or HA-MRSA status was included as the first input variable, followed by the inclusion of the test variable. The test variables were considered to be independently associated with the response variable when their regression coefficient was significant in multiple linear regression analysis. The significance threshold was set at 0.05 for all tests. The statistical analyses were performed by means of R software version 2.14 (The R Foundation for Statistical Computing, Vienna, Austria).

## Supporting Information

Table S1 **A comparison of distinct lineages of CA-MRSA and HA-MRSA with respect to cytotoxicity toward human osteoblasts, intracellular survival and alpha-toxin production.**
(PDF)
