# Supplementary material for: PSMs of Hypervirulent Staphylococcus aureus Act as Intracellular Toxins That Kill Infected Osteoblasts
Source: PLoS One. 2013 May 14;8(5):e63176. doi: 10.1371/journal.pone.0063176 (PMC3653922; doi:10.1371/journal.pone.0063176)
Supplement: Table S1 — A comparison of distinct lineages of CA-MRSA and HA-MRSA with respect to cytotoxicity toward human osteoblasts, intracellular survival and alpha-toxin production. (PDF) [file pone.0063176.s001.pdf]

Table S1. A comparison of distinct lineages of CA-MRSA and HA-MRSA with respect to cytotoxicity toward human osteoblasts, intracellular survival, and alpha-toxin production.

Lineage	LDH release by infected osteoblasts (mean n-fold change to 8325-4 strain [95%CI])	Viable intracellular bacterial load (mean n-fold change to 8325-4 strain [95%CI])	Alpha-toxin production (median ng/ml [IQR])
ST8-USA300-IV (n=5)	1.73 [1.40-2.07]	0.76 [0.38-1.14]	7650 [1110-9337]
ST80-IV (n=5)	1.71 [1.63-1.80]	0.99 [0.67-1.31]	1929 [1649-2434]
ST30-USA1100-IV (n=5)	1.56 [1.12-2.00]	0.42 [0.28-0.56]	5556 [5503-7715]
CA-MRSA (total, n=15)	1.67 [1.53-1.81]	0.72 [0.54-0.91]	5153 [1790-7683]
ST239-III (n=5)	1.06 [0.91-1.21]	2.70 [1.36-4.05]	2327 [143-3424]
ST228-I (n=5)	0.95 [0.87-1.03]	2.28 [1.58-2.98]	4 [3-8]
ST8-EMRSA2-IV (n=5)	0.92 [0.78-1.06]	2.84 [2.07-3.61]	2293 [305-3529]
ST22-EMRSA15-IV (n=5)	1.02 [0.85-1.18]	2.16 [1.48-2.85]	5729 [4193-5839]
HA-MRSA (total, n=20)	0.99 [0.93-1.04]	2.50 [2.15-2.84]	2310 [36-4326]
